# Loss Control-Based Key Distribution under Quantum Protection

**DOI:** 10.3390/e26060437

**Published:** 2024-05-22

**Authors:** Nikita Kirsanov, Valeria Pastushenko, Aleksei Kodukhov, Aziz Aliev, Michael Yarovikov, Daniel Strizhak, Ilya Zarubin, Alexander Smirnov, Markus Pflitsch, Valerii Vinokur

**Affiliations:** Terra Quantum AG, Kornhausstrasse 25, 9000 St. Gallen, Switzerland; vp@terraquantum.swiss (V.P.); ak@terraquantum.swiss (A.K.); aa@terraquantum.swiss (A.A.); mj@terraquantum.swiss (M.Y.); dst@terraquantum.swiss (D.S.); iz@terraquantum.swiss (I.Z.); asm@terraquantum.swiss (A.S.); pflitsch@terraquantum.swiss (M.P.)

**Keywords:** quantum cryptography, quantum communication, quantum-protected control-based key distribution, QCKD, loss control, long-distance fiber communication, optical time-domain reflectometry, OTDR, quantum networks, scalability of quantum protocols, non-orthogonal quantum states

## Abstract

Quantum cryptography revolutionizes secure information transfer, providing defense against both quantum and classical computational attacks. The primary challenge in extending the reach of quantum communication comes from the exponential decay of signals over long distances. We meet this challenge by experimentally realizing the Quantum-Protected Control-Based Key Distribution (QCKD) protocol, utilizing physical control over signal losses. By ensuring significant non-orthogonality of the leaked quantum states, this control severely constrains eavesdroppers’ capacities. We demonstrate the performance and scale of our protocol by experiments over a 1707 km long fiber line. The scalability of the QCKD opens the route for globally secure quantum-resistant communication.

## 1. Introduction

Quantum cryptography promising information transmission invulnerable to cyber threats stands at the forefront of a new era in secure communication. One of the major challenges here is the development of methods allowing for high-rate quantum communication over long distances. In real lossy quantum channels, particularly in the fiber-optic lines, transmissivity drops exponentially with distance, severely limiting the communication range. Despite this obstacle, remarkable progress has been achieved in extending quantum communication distances to hundreds and even a thousand kilometers [1]. Notable milestones in quantum communication include advancements in quantum one-time programs [2,3], quantum secure direct communication [4], the twin-field technique [1,5], measurement-device-independent quantum communication [6], satellite-based communication [7], and the time-bin technique [8,9]. Another possible way of extending transmission distances is the use of quantum repeaters [10,11,12,13,14,15], which are based on utilizing the quantum entanglement resource.

Recent theoretical papers [16,17] have established an alternative approach to overcoming the distance limitations of key distribution, which has later been realized experimentally [18]. The developed Quantum-Protected Control-Based Key Distribution (QCKD) follows the prepare-and-measure logics in the optical setting. In this protocol, the bits 0 and 1 are represented by the coherent states $|\gamma_{0}\rangle$ and $|\gamma_{1}\rangle$. The central idea is that the legitimate users Alice and Bob monitor the local signal leakages within the transmission channel, a fiber-optic line, and ensure that the leaked states—potentially captured by an eavesdropper, Eve—are substantially non-orthogonal. If the proportion of the leaked signal is $r_{E}$, then $\left|\langle \sqrt{r_{E}}\gamma_{0}|\sqrt{r_{E}}\gamma_{1}\rangle \right|\approx 1$, and as this scalar product closely approaches 1, the information accessible to Eve, constrained by the Holevo bound [19], goes to zero. As long as the leakage remains below a certain threshold, the users maintain an informational advantage over Eve, ensuring the safe distribution of the secret key. Importantly, given that the employed coherent states’ intensities, ${\left|\gamma_{0}\right|}^{2}$ and ${\left|\gamma_{1}\right|}^{2}$, are sufficiently low, eavesdropping on the homogeneously distributed Rayleigh scattering is unfeasible [16]. With that, the signal states can have sufficient intensities to be transmitted across a long fiber line containing optical amplifiers.

Here, we present the experimental realization of the QCKD protocol effectively working in a 1707 km long optical line. Our experimental settings include, in particular, specially designed bidirectional erbium-doped fiber amplifiers (BEDFAs) and a robust loss control apparatus. We show the precision and effectiveness of the loss control based on optical time-domain reflectometry (OTDR) [20,21]. As compared to [18], where experiments with a 1079 km line were reported, this study not only extends the protocol’s range but also addresses the impacts of statistical fluctuations and technical noise on the key distribution rate. Furthermore, here we demonstrate that the application of advantage distillation in the QCKD, which increases the system’s tolerance to errors, allowing for the accommodation of larger signal losses over longer transmission distances. Finally, we present the experimental results of the key distribution over various distances, including 1707 km. In addition, we discuss the possibility of expanding the QCKD to a multi-user network.

## 2. Description of the QCKD

In this section, we outline the implemented QCKD protocol, which is a modified version of the protocol described in detail in Ref. [16]. The protocol utilizes an optical fiber line for the transmission of quantum states and a classical authenticated channel for the exchange of service information.

Before beginning the main steps of the protocol, the users must complete an initialization phase. During this phase, and only during this phase, the users must be sure that the fiber line is secure against any unauthorized access or intervention by Eve. For instance, this phase may take place before deploying the fiber in the field. At this stage, the users establish the initial loss profile of the line by the OTDR method, which is widely used to assess the integrity of fiber lines, especially in the absence of optical amplifiers. The OTDR uses Rayleigh scattering as an instrument in detecting any new leakages that may arise along the fiber-optic line. The core principle involves transmitting a high-intensity probing pulse into the optical fiber, and then, measuring the power of the light that is backscattered to the source. This measurement records the distance to the corresponding scattering point, which is determined based on the time it takes for the light to return. Any newly occurring leakage changes the power of the backscattered radiation from the OTDR probing pulse. Specifically, the backscattered power decreases in the segment of the fiber where the eavesdropping intervention is located. The collected data from this process are represented in a reflectogram, which is a log-linear plot that depicts the backscattered power as a function of distance along the fiber.

Natural homogeneous losses, resulting from Rayleigh scattering due to density fluctuations in the fiber, cannot be effectively exploited by Eve, as described in detail in Ref. [16], and are considered as non-compromised losses. Conversely, localized losses, which occur at optical connections, splices, or bends where the signal can leak, can be eavesdropped. These losses must be either assumed to be compromised by Eve (and the users must take these losses into account in their evaluation of Eve’s information for the subsequent privacy amplification stage discussed below) or must be reduced by avoiding physical connectors and eliminating bends in the fiber. Our experimental analysis shows that losses at splice joints are minimal, around 0.1%, which is acceptable. Nevertheless, the use of continuous fiber without connections or splice joints further reduces the leakage.

In addition to assessing and pinpointing the losses, the OTDR also generates a unique fingerprint of the line. Given that quenched disorder within the fiber acts as a physically unclonable function, its corresponding reflectogram cannot be duplicated. Consequently, any interference into the fiber would alter this fingerprint, and thus, will be detectable [22]. The reflectogram obtained during initialization serves as the baseline fingerprint, allowing for the users to periodically use the OTDR for comparisons.

**Figure 1:**
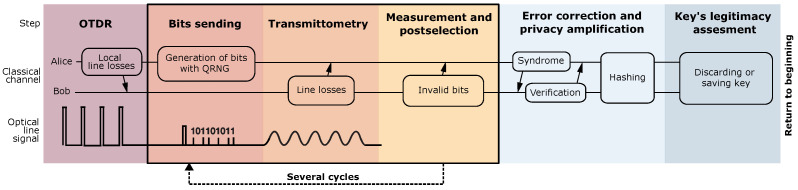
Main stages of the QCKD protocol. The information transmission between Alice and Bob over the classical authenticated channel is indicated by arrows.

The main steps of the protocol are depicted in Figure 1. The protocol starts with the OTDR scan, but this time, the users no longer need to ensure that Eve is absent. The inferred reflectogram is compared with the initial one. If the users observe a significant alteration in the line’s reflectogram, the protocol must be terminated, and the corresponding region of the line must be inspected for a breach.

After the OTDR phase, Alice encodes randomly generated bits into the phase-randomized coherent states, with 0 and 1 represented by different photon numbers carried by the light pulses. These photon numbers are not large, and as a result, these pulses’ states are non-orthogonal to each other. Alice sends a high-intensity synchronizing pulse every 31 bits, marking the beginning of a new bit package. This allows Bob to accurately identify the beginning of each bit package. On their side, Bob measures the energy of the received states to obtain bits.

Following the bit transmission, the process continues with the *transmittometry*. At this stage, Alice sends a high-intensity pulse with modulated intensity. Bob measures this pulse, and, by comparing the input and output power at the modulation frequency communicated over the authenticated classical channel, both users evaluate the total loss value. This value is then compared to the baseline to identify the extent of the newly appeared losses. The baseline for losses is updated in each OTDR session, providing the baseline value of the sine wave amplitude, $a_{\mathrm{ref}}$. Subsequently, by determining the signal amplitude, $a_{t}$, at the used frequency, the loss is calculated as $1-a_{t}/a_{\mathrm{ref}}$. The use of the high-frequency modulation reduces the impact of 1/*f* noise, which can distort the measurements; the applied method is, thus, similar to the lock-in technique [23]. If the loss control reveals losses exceeding some specific threshold $r_{c}$ established through a numerical optimization, it indicates that Eve may have gained excessive information about the bits. In such scenarios, users cannot maintain an information advantage over Eve, and must discard the affected bit package. However, if the losses remain below $r_{c}$, the users proceed with the postselection.

**Figure 2:**
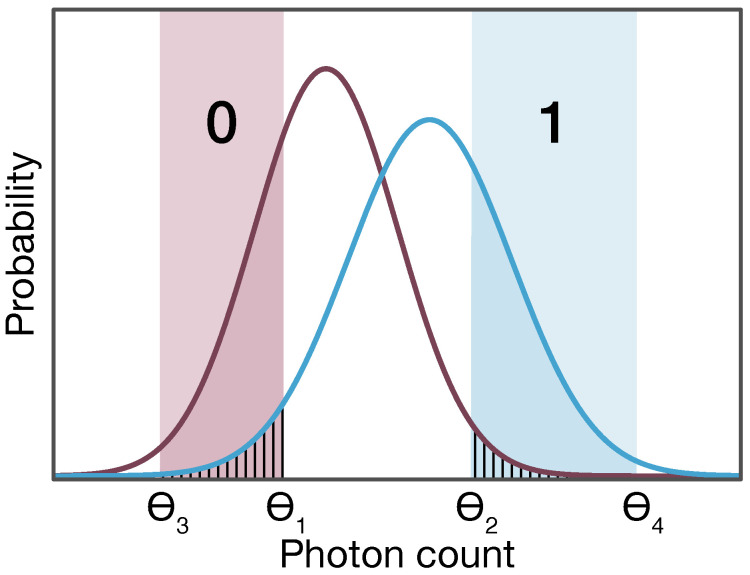
Postselection criteria. The measured photon counts within the range from $\Theta_{3}$ to $\Theta_{1}$ are designated as representing the 0 bit. Counts observed in the range from $\Theta_{2}$ to $\Theta_{4}$ are assigned the 1 bit. Bit positions corresponding to measurements outside these selected ranges are discarded.

Example probability distributions of the measured photon numbers for bits 0 and 1 are illustrated in Figure 2. The postselection process involves discarding bit positions based on unsatisfactory measurement outcomes. If the measured photon number falls between the expected average photon numbers for the 0 and 1 pulses, assigning it to either bit becomes challenging. Consequently, the users take photon numbers between $\Theta_{1}$ and $\Theta_{2}$ as inconclusive and discard those bit positions. Conversely, if the photon number measured by Bob is either too low or too high—making it straightforward to assign it to either 0 or 1—Eve might also easily deduce what these states represent. As explained in Ref. [16], this occurs because the use of optical amplifiers introduces correlations between the states that may have potentially leaked to Eve and the states measured at Bob’s end. Therefore, the users must also discard bit positions corresponding to photon numbers below $\Theta_{3}$ and above $\Theta_{4}$. Through this selective sifting of measurement results, the users generate the raw key.

To correct discrepancies between Bob’s bit string and Alice’s original sequence, the users execute the error correction procedure. In the experiment, we employ the low-density parity-check (LDPC) codes with advantage distillation, as explained in Section 6. After the error correction, the users perform the privacy amplification procedure to reduce Eve’s potential knowledge of the final key by compressing the distributed sequence. The compression coefficient is determined by the losses threshold $r_{c}$, which essentially puts the upper bound on Eve’s information about bits. In the experiment, we use a privacy amplification algorithm based on Toeplitz hashing [24].

The pulses’ and post-processing parameters and $r_{c}=2\%$ are chosen in such a way that if $r_{E}$ falls below $r_{c}$, then after post-processing Eve does not have any information about the resulting block of bits. This is based on the equation for $L_{f}$ (see Equation (1), which utilizes the users’ information advantage over Eve) [16]. Conversely, if $r_{E}$ exceeds $r_{c}$, the block is assumed to be compromised, and thus, is discarded. The binary decision making—to save or not to save a packet of bits—differs from the original approach of Ref. [16], which suggests adapting the pulses’ and post-processing parameters based on the leakage to harvest a useful key from every block of bits. However, the core principle of the protocol remains the same.

The security of the QCKD relies on the following additional physically justified assumptions [16]. First, it is assumed that the users’ loss control enables them to detect any replacement of a section of the quantum communication channel with another one. If such an eavesdropper’s action is detected, the users must terminate the protocol. Consequently, Eve must collect information about the optical signals propagating through the given channel, limiting Eve’s actions to those associated with that particular channel. The second assumption is that Eve cannot collect and effectively measure the natural signal losses occurring due to Rayleigh scattering, which are distributed homogeneously across the entire channel. As explained in Refs. [16,17], this would require constructing a device similar to a Maxwell demon, which must furthermore include an unfeasibly long antenna. Therefore, it is assumed that Eve is limited to creating local leakages or exploiting existing local leakages, such as losses at fiber bends or fiber connectors. All local losses can be detected by the users’ physical loss control, down to the loss control precision limit.

## 3. Experimental Setup

**Figure 3:**
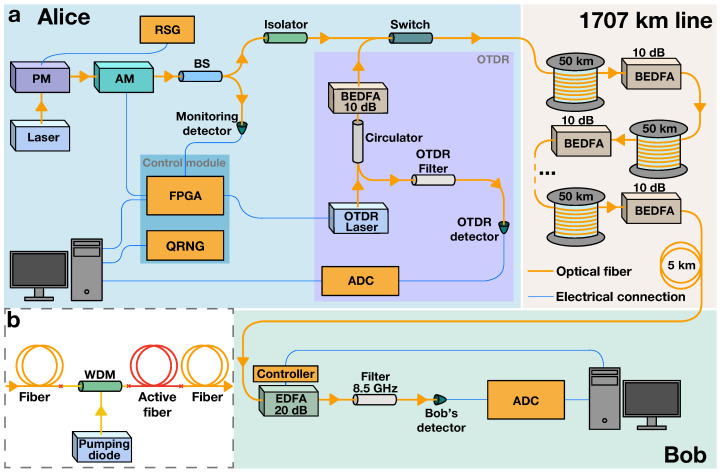
Experimental setup. (**a**) Schematics of the QCKD system. At Alice’s end, a phase modulator (PM) connected to a random signal generator (RSG) randomizes the phase of the light from the laser. A quantum random number generator (QRNG) creates a bit sequence, which is encoded into the passing light signal with a field-programmable gate array (FPGA) and an amplitude modulator (AM). A beam splitter (BS) redirects part of the signal to Alice for monitoring. The signal then passes through the 1707 km communication line, comprising 50 km fiber spans and bidirectional erbium-doped fiber amplifiers (BEDFAs). After preamplification, filtering, and detection at Bob’s end, the signal is converted to bits with an analog-to-digital converter (ADC). The switch alternates between key generation, and optical time-domain reflectometry (OTDR). (**b**) Scheme of the BEDFA (adapted from Ref. [16] under CC BY 4.0). A wavelength-division multiplexing (WDM) system merges the optical line’s signal with diode pumping into an active fiber segment, which then connects to the main fiber.

We begin by outlining our experimental QCKD setup. The setup is illustrated in Figure 3a. The key distribution begins with the generation of non-orthogonal optical states encoding random bits into coherent optical pulses at Alice’s side:

1. Coherent light from the 1530.33 nm laser source first passes through a phase modulator (PM) linked to a random signal generator (RSG), inducing the light’s phase randomization.
2. The light then enters a Mach–Zender amplitude modulator (AM), which forms bit-encoding optical states: The AM is linked to a control module comprising a quantum random number generator (QRNG) and a field-programmable gate array (FPGA). The FPGA converts *L* random bits from the QRNG into voltage pulses which are fed to the AM. The resulting light pulses corresponding to 0 and 1 comprise $|\gamma_{0}{|}^{2}=$ 10,000 photons and $|\gamma_{1}{|}^{2}=$ 10,600 photons, respectively.
3. The optical signal is split by a beam splitter (BS), with one part directed to Bob and the other part to a monitoring detector. The monitoring ensures precise adjustment of the control module and the AM. The primary signal portion then passes through an optical isolator to prevent noise and signal reflections from reaching the sending equipment.

The signal then travels through the 1707 km long transmission line, composed of the 50 km long optical fiber spans and BEDFAs. The main feature that distinguishes the BEDFA [16], depicted in Figure 3b, from the regular erbium-doped fiber amplifier (EDFA) [25,26] is the absence of optical isolators or circulators, allowing for the transmission of the backscattered components of the probing signal used for the OTDR. At Bob’s end, the signal undergoes several steps:

1. The signal is preamplified by 20 dB with an EDFA.
2. It then passes through a thermostabilized optical filter with an 8.5 GHz bandwidth, which eliminates noise in secondary modes caused by the amplifiers.
3. Finally, the signal reaches Bob’s detector. The analog signal from the detector is converted into a digital signal by an analog-to-digital converter (ADC).

The *L* bits now distributed between Alice’s and Bob’s computers undergo post-processing consisting of postselection, advantage distillation (explained in detail below), error correction, and privacy amplification.

Continuous monitoring of signal leakage $r_{E}$ is achieved using transmittometry and the OTDR:

- **Transmittometry.** During this phase, Alice’s AM and FPGA produce a high-intensity periodic signal at 25 MHz. Bob measures the signal at their end, and by comparing the input and output spectral power peaks, the users determine the total leakage in the line; the signal modulation suppresses the 1/f noise. Knowing the baseline of homogeneous natural losses (established during the preliminary stage without eavesdropping threats), the users can estimate the overall leakage.
- **The OTDR.** In this phase, the system activates the switch, halting the light transmission from Alice’s primary laser. The transmission line is then utilized for probing pulses generated by the OTDR module. A high-intensity probing pulse is produced by the dedicated OTDR laser controlled by the FPGA. The probing pulse is directed through an optical circulator, subsequently amplified by the BEDFA, and then, transmitted into the optical fiber line. The backscattered components then retrace their path back through the circulator and filter and are subsequently detected by the OTDR detector.

The signal wavelength of 1530.33 nm is specifically selected because it aligns with the peak of the BEDFA amplification gain spectrum in the C-band optical frequency range. Opting for a commonly used wavelength like 1550 nm would lead to a situation where the amplified spontaneous emission noise in the secondary modes—particularly around 1530 nm—would be amplified more than the actual signal. This can particularly result in radiation generation (the amplifiers would essentially work as lasers) and instability of the transmission line. Other details on BEDFAs can be found in Ref. [16].

It is also important to mention that the encoding scheme employed in our study is impervious to the effect of chromatic dispersion, which often leads to problems in long-distance optical signal transmission. As detailed in the Methods section, chromatic dispersion does not alter the photon counts in signal pulses, which carry the bit information. However, alternative schemes, such as encoding bits into the pulses’ shapes, may still be susceptible to chromatic dispersion, potentially resulting in an increased number of bit errors.

## 4. Optical Time-Domain Reflectometry

**Figure 4:**
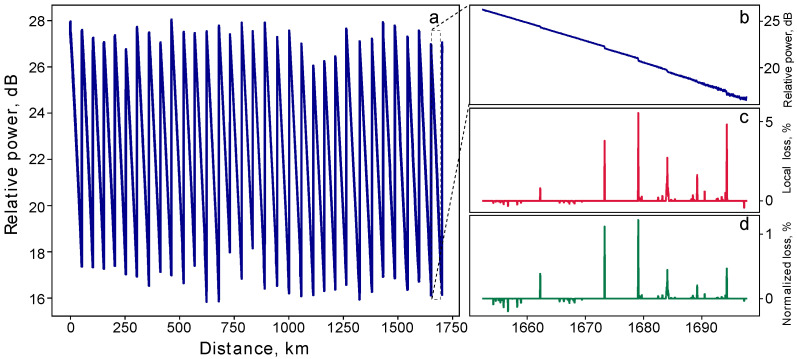
The OTDR leakage detection in the 1707 km long fiber line. (**a**) Detailed reflectogram of the entire 1707 km line, featuring 32 optical amplifiers. The measurements are conducted at a 1530 nm wavelength and with a probing-pulse duration of 200 ns; the reflectogram represents an average of 5000 individual probing-pulse runs. (**b**) Enhanced view of the 50 km segment near the end of the line: the reflectogram displays six local leakages (fusion splices), deliberately introduced to demonstrate the capacity of the loss control. (**c**) Loss profile for the same 50 km segment, illustrating the leakage measured in relation to the local power immediately preceding each leakage point. (**d**) Same loss profile, but with leakage quantified in relation to the initial input power.

We now discuss in detail the experimental loss control for the 1707 km line, concentrating on the OTDR [20,21]. In telecommunications, the OTDR is typically only applied to the segments of the transmission line that do not contain amplifiers. This is because standard amplifiers include elements, like optical circulators, that prevent the return of backscattered light, making it not possible to analyze the line in its entirety. However, our BEDFAs, a schematic of which is depicted in Figure 3b, are designed without these elements. Figure 4a displays the experimentally obtained reflectogram for the entire 1707 km line, including 32 BEDFAs. The measurement, which took 180 s (including both the physical measurements and the computational processing of the obtained data), was conducted at a wavelength 1530 nm, with a probing-pulse duration of 200 ns, and averaging over 5000 pulses’ runs. The reflectogram exhibits a characteristic saw-like pattern. This indicates the sharp increases in backscattered power at each BEDFA, providing a visual map of their positions along the fiber.

After obtaining the line’s reflectogram, we employ the $L_{1}$-filtering technique [27] to infer the loss profile of the line. This technique consists of approximating the reflectogram with a weighted sum of step-like functions (corresponding to local leakages) and a linear function (describing natural Rayleigh scattering losses) through minimizing the $L_{1}$ norm of the steps’ weights. Then, removing the linear part of the function and analyzing only the remaining step-like functions, we separate local leakages from the natural losses and background noise. Optical amplifiers additionally introduce step-like functions with positive discrete derivatives which must also be filtered out. After this filtering, we obtain the local losses from the remaining step-like functions by calculating the drops of these functions.

Panels (b) and (c) in Figure 4 illustrate the reflectogram and the loss profile (with leakage normalized to the power just before the corresponding scattering point), respectively, for a 50 km section subsequent to the 31st amplifier within our 1707 km line. The leakages, ranging from 1 to 5%, were purposefully induced in this specific segment to demonstrate the operation of the loss control. Figure 4d additionally presents the same loss profile but with leakage normalized to the initial power of the probing pulse, this loss profile is used for determining the value of $r_{E}$ utilized in the protocol. Despite the extensive length of the fiber, we achieved impressive OTDR precision, ranging from 0.01 dB at the beginning of the fiber section to 0.07 dB at the section’s end. We calculate this precision by constructing four individual reflectograms—each one representing an average over 1000 separate probe runs—and then, obtaining the standard deviation over these four reflectograms for each distance.

**Figure 5:**
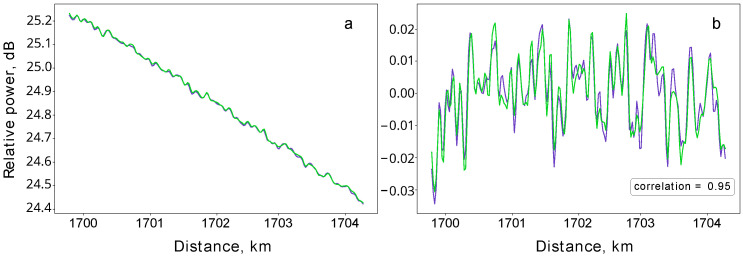
Unique backscattering patterns at 1700 km. (**a**) Two reflectograms (blue and green traces) correspond to successive measurements of the last 4 km long fiber section. Both reflectograms are obtained at a 1530 nm wavelength, with the duration of the probing pulse being 200 ns and averaging over 5000 pulses. (**b**) Subtracting the linear component of the reflectograms yields reproducible patterns of the backscattered signal, which correlate with a coefficient of 0.95.

Figure 5a presents two independently obtained reflectograms of the 4 km long fiber section at the end of the line. In turn, Figure 5b depicts the corresponding profiles obtained from the reflectograms by subtracting the linear components. Remarkably, the traces corresponding to the independent OTDR measurements exhibit identical patterns with a correlation coefficient of 0.95. This confirms that the observed features are not mere noise but are, in fact, unique patterns due to the amorphous structure of the silica fiber. Considering the significant distance of over 1700 km from the reflectometer, the consistency in replicating this pattern is highly notable. As the silica amorphous structure cannot be replicated, the observed distinctive pattern is a physically unclonable function [28,29,30,31]. Any physical tampering with the line would, thus, alter its characteristic ‘fingerprint’. With the BEDFAs maintaining the initial power levels of both the OTDR probing pulse and its backscattered components across the line, we can, thus, verify this unique ‘fingerprint’ and check the integrity of the fiber over the entire 1707 km line [22].

## 5. Statistical Fluctuations and Technical Noise

Experimental key distribution deals with finite data samples, whereas theoretical security proofs operate with probabilities. To bridge these two scenarios, the notion of the secret key and the security proof in the finite data regime can be properly modified [32]. In particular, this modification involves estimation of the upper bounds of the deviations of measurable statistical average values from their ideal expectation values and refining the formulas for the secure key rate to include experimentally measurable parameters derived from finite data sets. This approach requires introducing an additional parameter, a level of confidence that the actual secure key, obtained from the measured statistics, and the ideal key, based on probabilistic values, are indistinguishable with the given confidence level. Typically, the confidence level is selected to be 1−ϵ, with a pretty small value of $\epsilon ≃10^{-10}÷10^{-12}$, which corresponds to the tolerance interval with 6÷7 standard deviations. Below, we address the issue of discrepancy between the statistical mean values and the corresponding probabilities in our protocol.

Note that besides statistical fluctuations, there are additional noise sources that also impact the secret key rate. In the case of our protocol, the leakage $r_{E}$ is obtained via the loss control procedure that operates on a finite number of probing pulses, thus estimation of $r_{E}$ is influenced by the finite data statistics as well. Moreover, measurements at the loss control part are inherently noisy due to various technical imperfections, which further affects $r_{E}$. Security analysis of key distribution protocols typically focuses only on the statistical fluctuations due to the finite size of data used for key generation. In these protocols, all types of noise and imperfections in the communication line lead to an increased quantum bit error rate, or another metric indicating the potential presence of Eve. An important aspect of our protocol is that the secret key rate depends not only on the quantum data, but also on the line control procedure. The latter involves estimation of the local leakage $r_{E}$, which is affected by both statistical fluctuations and various technical imperfections. Following the theory of our protocol outlined in Ref. [16], the secret key rate depends on values $r_{E}$, $|\gamma_{0}{|}^{2}$, and $|\gamma_{1}{|}^{2}$. The secret key rate implies certain fixed values of $\gamma_{0/1}$ obtained through the secret key rate maximization, while in practice these values are derived from a calibration process that is inherently noisy due to the finite number of calibration measurements. The value $r_{E}$ is a free parameter that reflects the amount of eavesdropping, which we estimate by OTDR. We estimate the probability that our security will be compromised to be about $10^{-9}$, hence the expectation values $r_{E}$, $\gamma_{0}$, and $\gamma_{1}$ (utilized in the secret key rate formula) should fall within six standard deviations from the corresponding sample mean values. We also assume that all measurement results that are used for estimation of $r_{E}$, $|\gamma_{0}{|}^{2}$, and $|\gamma_{1}{|}^{2}$ are independent and identically distributed.

First, we start with the photon numbers $|\gamma_{0}{|}^{2}$ and $|\gamma_{1}{|}^{2}$. Ideally, these values are fixed, and can be found by optimization of the secret key rate for any given communication line. In practice, the pulses’ intensities are calibrated to a reference, which involves a finite number of intensity measurements. Specifically, we run a sequence of 50,000 calibration signals and gather the resulting voltage statistics from the detector. We then translate this statistics into photon numbers, and obtain the mean values and the standard deviations. The corresponding fluctuations surpass the shot noise due to the technical noise inherent to the electronics. To evaluate the impact of the finite statistics of the experimental dataset on the precision of the estimated values, we employ the previously mentioned assumptions. As an example, we obtain the sample mean values $|{\tilde{\gamma }}_{0}{|}^{2}=$ 9990 and $|{\tilde{\gamma }}_{1}{|}^{2}=$ 10,550 and standard deviations ${\tilde{\sigma }}_{0}=450$ and ${\tilde{\sigma }}_{1}=600$, which correspond to the estimates $|\gamma_{0}{|}^{2}={\left|{\tilde{\gamma }}_{0}\right|}^{2}-6{\tilde{\sigma }}_{0}/\sqrt{50,000}≃$ 9980 and $|\gamma_{1}{|}^{2}={\left|{\tilde{\gamma }}_{1}\right|}^{2}+6{\tilde{\sigma }}_{1}/\sqrt{50,000}≃$ 10,570.

Next, we estimate the value $r_{E}$ measured via the OTDR. Let the relative power levels of the light backscattered before and after the point of the local leakage be $D_{b}$ and $D_{a}$, respectively. We measure the difference ${\tilde{r}}_{E}=D_{b}-D_{a}$ by collecting 20 reflectograms each obtained by averaging over 5000 probing pulses. Based on the experimental data, we obtain the sample mean ${\tilde{r}}_{E}≃0.0146$ and standard deviation $\sigma_{r_{E}}≃5\cdot 10^{-4}$. To fall within the 6σ interval, the statistical estimate is $r_{E}={\tilde{r}}_{E}+6\sigma_{r_{E}}/\sqrt{20}≃0.0153$. After long-term monitoring of the communication line, we find that these are quite typical values. Thus, we set an upper bound $r_{E}=0.02$, which, according to our observations, is always satisfied.

**Figure 6:**
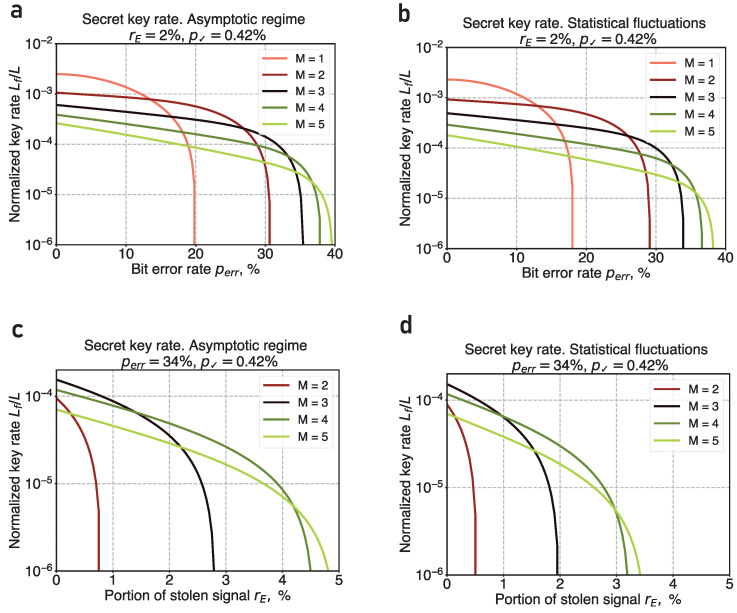
Secret key generation rate as a function of the observed parameters for different values of the block’s length *M*. We take that Eve is located right after Alice’s side, and the probability of a conclusive bit measurement result is *p*_✓_ = 0.42%. (**a**) Key rate dependence on BER ($r_{E}=2\%$), asymptotic limit. (**b**) Key rate dependence on BER ($r_{E}=2\%$), the statistical fluctuations are taken into account. (**c**) Key rate dependence on $r_{E}$ ($p_{\mathrm{err}}=34\%$), asymptotic limit. (**d**) Key rate dependence on $r_{E}$ ($p_{\mathrm{err}}=34\%$), the statistical fluctuations are taken into account.

In the next section, we utilize the calculated inaccuracies of $r_{E}$, $|\gamma_{0}{|}^{2}$, and $|\gamma_{1}{|}^{2}$ to compute the key rates (see Figure 6b,d) and perform the comparison with the asymptotic limit (Figure 6a,c).

## 6. Advantage Distillation and Final Key Length

Scattering losses and amplifier noise in a long transmission line raise the bit error rate (BER), $p_{\mathrm{err}}$, which in the case of our 1707 km line reaches as much as 34.0%. Under such conditions, the standard error correction based on the low-density parity-check (LDPC) codes and privacy amplification are insufficient for securing a key against potential adversaries. To increase the tolerance to errors and harvest the key even with $p_{\mathrm{err}}=34.0\%$, we employ the so-called advantage distillation technique [33,34,35,36].

The advantage distillation is conducted after the postselection but before the error correction stage. During this phase, the legitimate users divide their sifted strings into blocks of length *M*. For each block $a\in {\left\{0,1\right\}}^{M}$, Alice publicly declares a syndrome of the block according to the repetition code with the block’s length *M*. In this way she announces that her block is either *a* or $\bar{a}$ (the overline stands for the inversion of *a*). If Bob’s corresponding block coincides with *a* or $\bar{a}$, the users agree to count it as a single bit of the raw key (e.g., *a* means 0, $\bar{a}$ means 1), otherwise it gets discarded. The probability for a block to survive this stage is equal to $p_{\mathrm{err}}^{M}+{\left(1-p_{\mathrm{err}}\right)}^{M}$, while the modified error probability in the new raw key is ${\tilde{p}}_{\mathrm{err}}=p_{\mathrm{err}}^{M}/\left(p_{\mathrm{err}}^{M}+{\left(1-p_{\mathrm{err}}\right)}^{M}\right)$. For more details on the technique, see Methods section.

Following the advantage distillation, Alice and Bob carry out the standard error correction and privacy amplification procedures, obtaining the final secret key. If *L* is the number of bits that Alice initially sends to Bob, the length of the final key is

(1) $$L_{f}=\frac{p_{✓}L}{M}\left(p_{\mathrm{err}}^{M}+{\left(1-p_{\mathrm{err}}\right)}^{M}\right)\cdot \left[1-f\left({\tilde{p}}_{\mathrm{err}}\right)h_{2}\left({\tilde{p}}_{\mathrm{err}}\right)-I\left(A:E\right)\right],$$

where *p*_✓_ is the proportion of bits that are not discarded during the postselection, and $(p_{\mathrm{err}}^{M}+{\left(1-p_{\mathrm{err}}\right)}^{M})/M$ corresponds to the bit reduction due to the advantage distillation. The term in the square brackets results from the Devetak–Winter equation [37] and represents the informational advantage of Alice and Bob over Eve: the classical information disclosed to Eve during error correction is $f\left({\tilde{p}}_{\mathrm{err}}\right)h_{2}\left({\tilde{p}}_{\mathrm{err}}\right)$, where the parameter f≥1 characterizes the efficiency of a particular correction code, $h_{2}$ is the binary entropy, and I(A:E) is the information that Eve can extract from the intercepted proportion of signal (see Equations (3) and (4) in Methods).

Figure 6 shows the final key generation rate in the presence of advantage distillation as a function of the observed parameters. This is illustrated in two scenarios: one in the asymptotic limit (see Figure 6a) and the other accounting for finite-size effects and fluctuations (see Figure 6b). The five curves in each plot represent different values of the block’s length *M*; M=1 corresponds to the scenario without advantage distillation. In the asymptotic limit, the system’s tolerance to the BER can be improved from 20% up to 39%, while in the finite-size case, it reaches 37%. However, this increase in the BER tolerance comes at a cost, notably reducing the key generation rate by two orders of magnitude. Such high critical BER values are achieved for M=5; further increase in *M* provides a slight improvement in the critical BER, yet, it leads to a much more significant decrease in the key rate.

The advantage distillation also significantly enhances the system’s resilience against the leakage $r_{E}$. Figure 6 shows the final key distribution rate as a function of $r_{E}$ for the fixed BER value of $p_{\mathrm{err}}=34\;\%$, as well. Here, Figure 6c,d correspond to the asymptotic and finite-size regimes, respectively. It is important to note that for $p_{\mathrm{err}}=34\;\%$ the secure key distribution becomes unattainable without advantage distillation. Nevertheless, advantage distillation with any non-trivial block length (M≥2) solves this problem and yields non-negative secret key rates. Particularly, with a block length of M=5, successful key distribution is achievable for leakage rates as high as 4.8% in the asymptotic limit and up to 3.4% in the finite-size scenario.

Our results add to the findings established in our previous publications [17,38], in which we explored various strategies to enhance the efficiency of key distribution through modifications of post-processing.

## 7. Key Distribution Results

**Figure 7:**
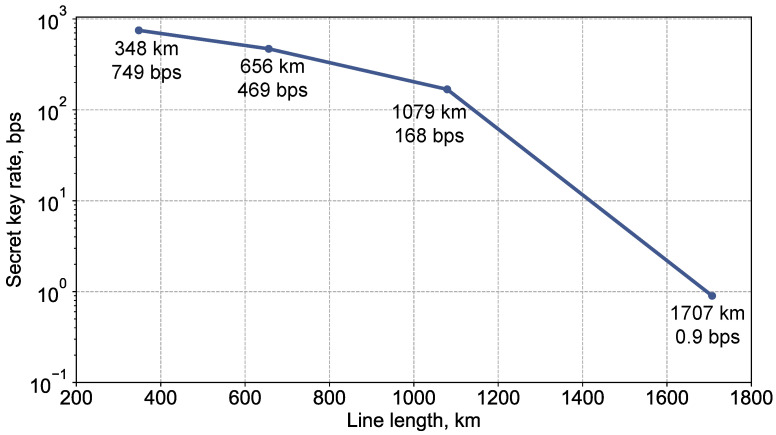
Graphical representation of the key rates achieved at various transmission distances using our experimental setup. The key rate of 0.9 bps is achieved over the 1707 km line. Additional key rates for other transmission lengths are obtained with similar equipment. All results correspond to the asymptotic limit.

We now present the results of the key distribution itself. With the parameters $r_{c}=2\%$, $|\gamma_{0}{|}^{2}=10,000$, and $|\gamma_{1}{|}^{2}=10,600$, leveraging a random number generation rate of 5 Mbps at Alice’s side, and advantage distillation with a block size of M=3, we achieve a key rate of 0.9 bps across our 1707 km optical fiber line. This assumes that the value of $r_{E}$ is below $r_{c}$, as ensured by the loss control precision. This achievement, along with the key rates obtained at other transmission distances using the same setup, is depicted in Figure 7 (all results are obtained in the asymptotic limit). We note that we did not encounter radiation generation, so despite comprising the 32 BEDFAs, the line remains stable.

To assess the statistical quality of the distributed bits, we accumulate them over an extended period of time and divide them into 2930 segments, each containing 1024 bits. We then compute the average and variance of the bit sum per segment across the entire ensemble, yielding 512.4 and 255.9, respectively. This result closely aligns with the theoretical expectations of 512 and 256, corresponding to the binomial distribution. Such consistency with the binomial distribution additionally underscores the robustness of the realized QCKD. Further analysis of the key’s statistical properties, including a comprehensive examination with NIST testing methods, will be uncovered in our upcoming publication.

## 8. The QCKD Network

Beyond the point-to-point secure communication, the expanding digital landscape demands the quantum-resistant key distribution solutions for multiple users [39,40,41,42,43,44,45,46]. It turns out that the operational principle of our QCKD opens the possibility of building a zero-trust key distribution network that does not require connecting everyone to everyone. This section outlines such a network architecture, where users can alternate between receiving and transmitting modes under the supervision of other network participants.

**Figure 8:**
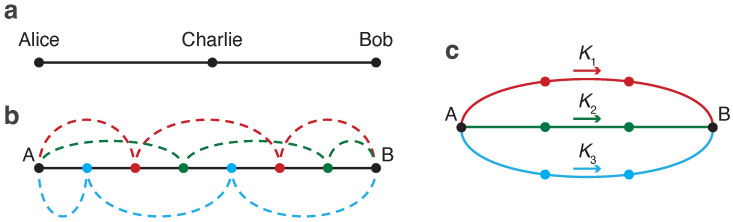
Simplest QCKD networks. (**a**) Tripartite network where the users (Alice, Bob, and Charlie) are linked by a single quantum channel. Alice can send a key to either Bob or Charlie. Depending on who is the intended recipient, Charlie needs to alternate between receiving and transmitting modes (under the watchful eye of Alice and Bob). (**b**) Within a linear chain of users, User A has the option to transmit keys to User B through various sets of reproducers. These sets, colored red, green, and blue, provide different pathways for the keys. (**c**) A network structure equivalent to (**b**). User A distributes different initial keys ($K_{1}$, $K_{2}$, and $K_{3}$) through the red, green, and blue sets of reproducers, respectively. As these sets are mutually exclusive, no single reproducer has knowledge of the entire final key $K=H\left[K_{1},K_{2},K_{3}\right]$.

Consider a network with three users, Alice, Charlie, and Bob, connected via a direct quantum channel; see Figure 8a. Each pair of users is also linked via a classical authenticated channel, which is not depicted. Each user is equipped with a switch that enables the reception of signals through this channel or the bypassing of signals to the intended receiver. When Alice initiates a signal transmission to their adjacent neighbor, Charlie, they must fully capture and measure it, blocking the next participant, Bob, from this transmission. Alternatively, if Alice targets their signal for Bob, Charlie’s switch must be configured to direct the signal exclusively to Bob. A critical aspect of the proposed communication system is the ability of the users to monitor channel losses end-to-end. This allows Alice and Bob to ascertain that Charlie does not intercept—or even partially access—the signal when it is not designated for him. Upon detecting any unauthorized intervention by Charlie, such as signal tapping, the transmission is immediately terminated, and the affected bits are discarded. The classical authenticated channels enable user coordination, allowing them to decide who will participate in the key distribution and in what order.

While it is feasible for the sender to transmit the key directly to the recipient, bypassing all intermediate nodes, this approach may not always be viable due to the decrease in the key rate with increasing distance. Therefore, instead of bypassing all intermediate users, it may be more advantageous to bypass only some of them. The others would then act as trusted nodes, measuring the signal and resending it; we will refer to these users as *reproducers*. In this scenario, the sender and recipient may employ the secret sharing scheme [47,48,49]. Consider the one-dimensional network depicted in Figure 8b. Within this user chain, three different initial keys, $K_{1}$, $K_{2}$, are $K_{3}$, are distributed via different sets of reproducers, marked as red, green, or blue. By controlling leakages across the entire line—either collectively or through some central entity that does not have access to the keys themselves—it is ensured that only the designated group of reproducers handles a specific initial key. Thus, while each group of reproducers knows their respective $K_{i}$, they lack knowledge of the other initial keys. The final key, *K*, is composed of all initial keys (e.g., through a hashing algorithm, $K=H\left[K_{1},\;K_{2},\;K_{3}\right]$) and remains unknown to any of the reproducers. The introduced control mechanism effectively increases the connectivity of the QCKD network: what was initially a simple one-dimensional user chain in Figure 8b becomes equivalent to a more interconnected network with multiple branches, as illustrated in Figure 8c.

The design of the switches remains a subject of future investigation. Key requirements for such switches include minimal local leakage and the capability for extreme transmittivity control—meaning the switch should either fully transmit or completely block the signal. Potential designs for these switches may encompass configurations like the Mach–Zehnder interferometer, incorporating beam splitters and at least one phase modulator, as well as other optical elements such as microelectromechanical systems (MEMSs), lithium niobate (LiNbO_3_), and optomechanical components.

## 9. Discussion

In this study, we have demonstrated the practical implementation and performance of the QCKD over an extensive 1707 km fiber line. We have shown the robustness of the system’s various components, in particular, the effectiveness of the used equipment, including the BEDFAs and loss control and advantage distillation procedure. These developments collectively enhance the scalability of the QCKD solution, enabling quantum-protected communication over unprecedented distances. Furthermore, we have discussed the potential of the QCKD in multi-user key distribution, paving the way for broader applications. The subject of QCKD networks will be further studied in our upcoming publication.

A notable feature of the keys generated through the QCKD is their everlasting security, which is inherent to quantum cryptography [50]. Unlike classical cryptography, once the keys are securely distributed through the QCKD they remain impossible to compromise by attacking the QCKD hardware, including the loss control components. Our findings establish the viability of device-dependent quantum cryptography and underscores its remarkable capacity.

## 10. Methods

### 10.1. Chromatic Dispersion Effect

Long-distance transmission of optical signals usually faces the problem of chromatic dispersion [51]. Chromatic dispersion is the variation in the refractive index of an electromagnetic wave across different optical frequencies. When an optical pulse propagates, it travels at the group velocity, but the phenomenon known as a group-velocity dispersion (GVD) contributes to the widening or broadening of the pulse. The effect blurs the bit encoding wave packets so the adjacent ones partially overlap. In the case of key distribution, this may cause additional errors and limit the key rate [52,53,54].

We estimate the influence of chromatic dispersion on a single pulse, which is presented in the form of a super-Gaussian pulse at the beginning of the transmission line. The amplitude’s dependence on time *T* for a super-Gaussian pulse is proportional to $\exp(-{\left(T/4T_{0}\right)}^{2m})$. In our case, m=2 and $T_{0}\approx 1.2$ ns. The relative time broadening of such a pulse can be calculated according to the following equation; see chapter 3 in Ref. [51]:

(2) $$\alpha =\sqrt{1+m^{2}\frac{\Gamma (2-1/2m)}{\Gamma (3/2m)}{\left(\frac{\beta_{2}z}{T_{0}^{2}}\right)}^{2}},$$

where Γ(·) is the gamma function, *z* is the transmission distance, and $\beta_{2}$ is the GVD constant. In our case of 1530 nm wavelength, $\beta_{2}=-19.7\;{\mathrm{ps}}^{2}/\mathrm{km}$. The resulting broadening α at a distance of 1707 km is only 0.08% of the initial time width and can be considered insignificant compared to the time interval between adjacent pulses, which is about 5 ns.

**Figure 9:**
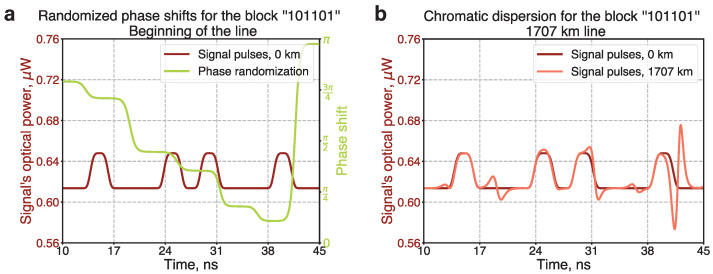
Numerical simulations of the influence of chromatic dispersion on phase-randomized signal pulses propagating along a 1707 km line. (**a**) Temporal profile of signal’s optical power for a bit sequence “101101” (dark red curve) and temporal profile of randomized phase shift (green curve). The time duration of each bit is 2 ns. The optical power for bits “0” and “1” corresponds to average photon numbers of 10,000 and 10,600, respectively. (**b**) Temporal profile of the signal’s optical power, influenced by chromatic dispersion (orange curve), at a distance of 1707 km compared to the initial temporal profile. The shape is modified at moments when the phase shift switches from one value to another.

In the case of phase randomization, chromatic dispersion significantly alters the temporal shapes of propagating pulses. We conduct numerical simulations to examine the evolution of bit-encoding wave packets for a representative sequence of phase-randomized pulses. Figure 9a illustrates the temporal profile of optical pulses corresponding to the bit sequence ‘101101’ and randomized phase shifts. The amplitude of the initial signal is multiplied by a phase factor determined by the green curve. After propagating through the whole 1707 km line, the shapes of the pulses are altered at moments corresponding to the phase shift switching, as shown in Figure 9b. However, the total number of photons (or energy) in each pulse remains constant.

Consequently, in the case of the photon number encoding scheme implemented in this experiment, chromatic dispersion does not impact the key distribution, as the modification of the pulses’ temporal profiles does not influence the measurement results. However, in the scenario of shape coding—where bits are encoded into the temporal profiles of the pulses—the legitimate users should either refrain from phase randomization or employ methods to mitigate chromatic dispersion [55].

### 10.2. Advantage Distillation Specifics

**Figure 10:**
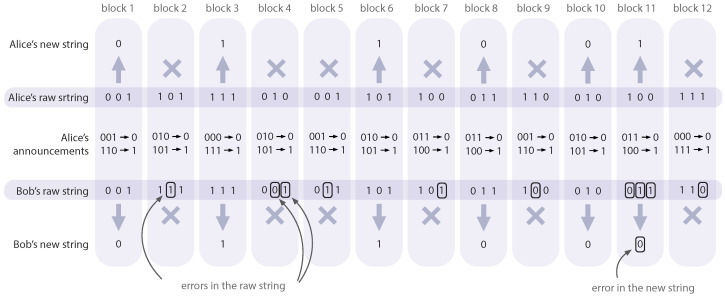
Schematic of the advantage distillation procedure. Alice divides the raw string into blocks of length *M* (here, M=3). For every block, Alice publicly announces two pieces of information: the block’s actual value and its bitwise inverse (without telling which one is which). If Bob’s block does not match either of the values Alice announced, both Alice and Bob discard that particular block (the discarded blocks are marked as “×”). The remaining blocks are translated into a new bit string with a lower BER (we mark a new bit which is incorrect).

The advantage distillation procedure is schematically shown in Figure 10. The example raw string of a length 36 bits is divided into 12 blocks. For each block *a*, Alice announces *a* and $\bar{a}$ and the rule according to which blocks will be translated to the bits of the new string. If Bob’s block coincides with *a* or $\bar{a}$, it is transformed into one bit of a new string (see 2nd, 3rd, 6th, 8th, 10th, and 11th blocks in Figure 10), otherwise it is discarded. The length of the blocks, *M*, should be chosen in accordance with the observed parameters (the raw BER, $p_{\mathrm{err}}$, and leakage, $r_{E}$) to achieve the maximally attainable key rate.

In the most general case, Eve’s information I(A:E), appearing in Equation (1), depends on the values of the pair of blocks between which the eavesdropper has to distinguish, $\left\{a,\bar{a}\right\}$. The estimate of the intercepted information is given by the maximal value of the Holevo bound [19] for all the ensembles corresponding to different blocks *a*:

(3) $$I\left(A:E\right)\leq \max_{a\in {\left\{0,1\right\}}^{M}}\chi \left(E_{a}\right),$$

where Eve’s ensemble, conditioned on Alice having announced block *a*, is the following:

(4) $$E_{a}=\left\{\left(\frac{1}{2},\;\rho_{E}^{\left(a_{1}\right)}⊗\ldots ⊗\rho_{E}^{\left(a_{M}\right)}\right)\right.,\;\left.\left(\frac{1}{2},\;\rho_{E}^{\left({\bar{a}}_{1}\right)}⊗\ldots ⊗\rho_{E}^{\left({\bar{a}}_{M}\right)}\right)\right\}.$$

Here, $a_{i}$ stands for the *i*-th bit of block *a* and $\rho_{E}^{\left(a_{i}\right)}$ is Eve’s density matrix for this single bit $a_{i}$. Such an ensemble appears when Eve possesses quantum memory and can maintain intercepted states until blocks’ announcements.

The density matrix of Eve for a single bit is influenced by the position of the leakage and the state coming from Alice’s end. In this work, we consider that the adversary’s intrusion occurs immediately after Alice. Ideally, Alice prepares a pure coherent state with an amplitude $\gamma_{a_{i}}$ and randomizes its phase. However, in our experimental setup, the electronics controlling the amplitude modulation of the generated states (refer to Figure 3) do not provide a predetermined value of controlling voltage for a fixed bit of a key. Consequently, for a given bit $a_{i}$, Alice prepares a mixture of phase-randomized coherent states with a probability distribution $p_{a_{i}}\left(|\gamma |\right)$, which is determined by the electronics in the amplitude modulator. The density matrix of the eavesdropper’s subsystem can be written as follows:

(5) $$\begin{array}{cc} \rho_{E}^{\left(a_{i}\right)}=\sum_{\left|\gamma \right|}p_{a_{i}}\left(|\gamma |\right)\frac{1}{2\pi }\int_{0}^{2\pi }d\varphi \;\;|e^{i\varphi }\sqrt{r_{E}}\left|\gamma \right|\rangle & \langle e^{i\varphi }\sqrt{r_{E}}\left|\gamma \right||E\\ & =\sum_{\left|\gamma \right|}p_{a_{i}}\left(|\gamma |\right)e^{-r_{E}{\left|\gamma \right|}^{2}}\sum_{n=0}^{+\infty }\frac{{\left(r_{E}{\left|\gamma \right|}^{2}\right)}^{n}}{n!}|n\rangle \;{\langle n|}_{E}. \end{array}$$

Substituting Equations (4) and (5) into Equation (3), we estimate the upper bound of the eavesdropper’s information. The estimation is in turn utilized for computing the secret key generation rate according to Equation (1); the results are depicted in Figure 6.

## Data Availability

The supporting data for the findings drawn in this research are present within the main article. The source data can be obtained from the authors upon a request.

## Abbreviations

The following abbreviations are used in this manuscript:

ADC	Analog-to-digital converter
AM	Amplitude modulator
BEDFA	Bidirectional erbium-doped fiber amplifier
BER	Bit error rate
BS	Beam splitter
EDFA	Erbium-doped fiber amplifier
FPGA	Field-programmable gate array
GVD	Group-velocity dispersion
MEMS	Microelectromechanical system
OTDR	Optical time-domain reflectometry
PM	Phase modulator
QCKD	Quantum-Protected Control-Based Key Distribution
QRNG	Quantum random number generator
RSG	Random signal generator
WDM	Wavelength-division multiplexing
